# Large Language Models and Communication of Medical Probabilities

**DOI:** 10.1001/jamanetworkopen.2025.50449

**Published:** 2025-12-17

**Authors:** Nicholas J. Jackson, Katerina Andreadis, Jessica S. Ancker

**Affiliations:** 1 Department of Biomedical Informatics, Vanderbilt University Medical Center, Nashville, Tennessee; 2 Department of Population Health, NYU Grossman School of Medicine, New York, New York

## Abstract

This diagnostic study investigates how chatbots interpret probability terms, such as rare, common, and possible, when describing medical risk.

## Introduction

Verbal probability terms (eg, *rare* or *common*) are used to communicate medical risks. Patient interpretations of such terms vary widely and differ from standardized definitions, such as the European Commission (EC) guidelines, leading to potential misunderstandings.^1^ Large language model (LLM) chatbots have been hypothesized to improve health care communication^2^ and are increasingly used as sources of health advice.^3^ However, because LLMs embed the biases present in large corpora of human-generated texts,^4^ this study hypothesized that LLMs emulate lay interpretations of verbal probability terms, potentially exacerbating patient misunderstandings of medical risks. This study assessed how commercial LLMs interpret verbal probability terms used to communicate medical risks.

## Methods

For this diagnostic study, we selected 4 commonly used LLMs: ChatGPT-4o (LLM 1), Gemini 2.0 (LLM 2), Grok 2.0 (LLM 3), and Claude 3.5 Sonnet (LLM 4). Between March 10 and 13, 2025, we simulated patient questions, prompting these LLMs to interpret 10 verbal probability terms identified in our prior meta-analysis.^1^ We varied the severity of the clinical condition being asked and the presence of anxious language in the question.

Experiment 1 assessed how LLMs respond to naively written patient questions (ie, without deliberate prompting techniques). We measured how often the LLMs failed to provide a numeric interpretation (abstention rate) and the length and readability of their responses (Flesch Reading Ease). Experiment 2 modified the system message to require the LLMs to provide a numeric interpretation in the form of a single probability (ie, to not abstain). This both simulated how a more experienced user might prompt LLMs and ensured the completeness of the LLMs’ responses for our analysis. We followed the TRIPOD reporting guidelines and provided all materials necessary to reproduce this study online in the eReference in Supplement 1. This study did not involve human participants, as defined by 45 CFR §46, and therefore did not undergo institutional review board review or require informed consent. Statistical significance was calculated using mixed-effects logistic regression, 2-way mixed analysis of variance, and 1-sample *t *tests with significance set at *P* <. 05. Prompts to the LLM were made using Python 3.12 and statistical analysis was performed using R version 4.3.3 (R Project for Statistical Computing). Additional details on the prompting strategy and statistical analysis are available in the eMethods in Supplement 1.

## Results

The abstention rates for each LLM varied widely (Table 1) and were increased by both clinical severity (odds ratio [OR], 3.25; 95% CI, 2.46-4.30; *P* < .001) and anxious wording (OR, 4.63; 95% CI, 3.47-6.19; *P* < .001). These associations varied by LLM (*P* for interaction between LLM and severity = .008; *P* for interaction between LLM and anxiety = .001). Anxious wording and clinical severity were associated with increased response length by 69% and 15%, respectively. Similarly, anxious wording and clinical severity raised the Flesch Reading Ease scale by 3.7 points (95% CI, 2.5-5.0 points; *P* < .001) and 2.0 points (95% CI, 0.7-3.3 points; *P* = .011), respectively, indicating higher readability.

**Table 1.** Abstention Rates of Different LLMs, Stratified by Severity and Anxiety

LLM (n = 800 prompts each)	Abstention rate, mean (95% CI), %			*P* value
	Overall	High severity	Anxiety	
1	93.5 (91.6-95.0)	98.2 (96.4-99.1)	98.5 (96.8-99.3)	NA
2	93.0 (91.0-94.6)	95.2 (92.7-96.9)	94.2 (91.5-96.1)	.69
3	76.1 (73.1-78.9)	86.5 (82.8-89.5)	91.0 (87.8-93.4)	<.001
4	30.9 (27.8-34.2)	38.5 (33.9-43.4)	42.8 (38.0-47.6)	<.001

Abbreviations: LLM, large language model; NA, not applicable.

When LLMs provided numeric interpretations, they aligned closer to lay expectations than EC regulatory guidance (Table 2). For example, all LLMs overestimated the EC usage of common, which has an upper limit of 10% (LLM 1: 38.5%, 95% CI, 34.4%-42.5%; LLM 2: 65.5%, 95% CI, 63.1%-67.9%; LLM 3: 24.5%, 95% CI, 22.1%-26.9%; LLM 4: 16.5%, 95% CI, 15.0%-18.0%; *P* < .001). Moreover, 33 of 40 LLM × verbal term pairs changed significantly due to anxious wording or clinical severity, as assessed by 1-way ANOVA.

**Table 2.** Numeric Interpretations of Verbal Probability Terms From Prior Meta-Analysis, EC Guidance, and LLMs

Verbal term	Human (meta-analysis), mean (95% CI), %^a^	EC guidelines, range, %^b^	Mean (SD), %				
			LLM mean^c^	LLM 1	LLM 2	LLM 3	LLM 4
Rare	10.00 (7.99-12.01)	0.01-0.10	4.2 (2.3)	4.9 (1.7)	6.0 (2.1)	1.6 (1.5)	4.2 (1.3)
Uncommon	17.60 (13.19-22.09)	0.10-1.00	11.8 (3.3)	10.6 (4.4)	12.0 (2.5)	9.5 (1.5)	15.0 (0.0)
Unlikely	17.70 (14.86-20.55)	NA	19.2 (3.8)	18.0 (5.0)	20.0 (0.0)	18.5 (3.7)	20.5 (4.3)
Possible	43.30 (36.66-49.89)	NA	30.0 (17.3)	24.7 (11.1)	55.5 (5.1)	13.8 (6.3)	26.2 (7.2)
Common	58.70 (50.40-67.06)	1.00-10.00	36.2 (19.6)	38.5 (8.6)	65.5 (5.1)	24.5 (5.1)	16.5 (3.3)
Very common	60.10 (42.36-77.85)	≥10.00	64.8 (16.5)	67.2 (11.5)	70.0 (0.0)	80.0 (0.0)	41.8 (12.7)
Probable	69.90 (67.07-72.67)	NA	69.5 (5.8)	69.8 (2.6)	75.0 (0.0)	63.0 (6.2)	70.2 (4.4)
Likely	71.90 (69.90-73.84)	NA	69.1 (5.9)	67.5 (5.3)	74.2 (1.8)	66.8 (7.7)	67.8 (4.1)
Usual	75.40 (71.53-79.23)	NA	42.0 (16.6)	50.5 (8.7)	50.0 (0.0)	52.5 (4.4)	15.1 (5.7)
Very Likely	84.30 (79.43-89.17)	NA	82.2 (5.8)	77.0 (4.1)	90.0 (0.0)	82.8 (2.6)	79.0 (3.5)

Abbreviations: EC, European Commission; LLM, large language model; NA, not applicable.

^a^ Denotes random-effects meta-analytic means and 95% CIs from the published systematic review of human estimates.

^b^ Denotes EC labeling guidance for adverse-event frequencies. Terms not covered by the guideline are denoted using NA.

^c^ LLM figures are means (SD) across baseline (mild, nonanxious) responses per model.

## Discussion

In this diagnostic study, LLMs frequently abstained from providing numeric interpretations of verbal probability terms, against previously reported patient preferences.^1^ When LLMs did not abstain, they aligned closer to lay interpretations than clinical guidelines, suggesting that patient-facing LLMs may amplify misunderstandings of medical risks. Moreover, LLMs’ abstention rate, readability, and word count increased with anxious wording and clinical severity. This increased abstention rate is concerning as such patients are more likely to seek online health information.^5^ However, increased length and readability have been highlighted as a strength of LLMs,^6^ suggesting that LLMs could be adaptive to individual patient preferences under anxiety or increased clinical severity.

The main limitations of this study are that our quantitative analysis may have omitted nuances in LLM responses and that the prompts we used may not perfectly capture how patients communicate with LLMs. In conclusion, LLMs inconsistently interpret verbal probability terms used to communicate medical risks. Additional research into technical guardrails is needed before LLMs can effectively communicate medical risks.

## Notes

Supplement 1. eMethods. Prompt Template eReference. Code Repository

Supplement 2. Data Sharing Statement
