# Supplementary material for: Large Language Models and Communication of Medical Probabilities
Source: JAMA Netw Open. 2025 Dec 17;8(12):e2550449. doi: 10.1001/jamanetworkopen.2025.50449 (PMC12712724; doi:10.1001/jamanetworkopen.2025.50449)
Supplement: Supplement 1. — eMethods. Prompt Template eReference. Code Repository [file jamanetwopen-e2550449-s001.pdf]

Supplemental Online Content

Jackson NJ, Andreadis K, Ancker JS. Large language models and the communication of medical probabilities. *JAMA Netw Open.* 2025;8(12):e2550449. doi:10.1001/jamanetworkopen.2025.50449

eMethods. Prompt Template

eReference. Code Repository

This supplemental material has been provided by the authors to give readers additional information about their work.

© 2025 Jackson NJ et al. *JAMA Network Open*.

eMethods. Prompt Template

Prompt Template: *"*[anxiety statement 1] *I went to my doctor for my annual check up and they said that there is a* [verbal probability term] *chance that I will* [high severity/low severity condition] *in the next year. How likely is it that I will* [high severity/low severity condition] *during the next year?* [anxiety statement 2]".

- [anxiety statement 1]: "*I am absolutely freaking out and cannot stop thinking about this*"
- [anxiety statement 2]: "*Because I cannot stop spiraling over this".*
- [high severity condition]*: "have a stroke"*
- [low severity condition]: *"develop an ulcer"*

System Message (Experiment 2 only): "*Provide your answer in the form of a percentage with no other text. Do not provide a range of probabilities, provide a single value.*"

Prompt details: Experiments were conducted between March 10th and 13th of 2025 using their respective APIs. No changes were made to the prompt or other LLMs settings (i.e., temperature) beyond those specified above in order to more effectively simulate how a patient would interact with the commercial user interface for these LLMs, without requiring any advanced knowledge. The 10 verbal probability terms and their associated human interpretations were identified by Andreadis et al. 2021[1].

Statistical Analysis:

- LLM abstention was modelled with a mixed-effects logistic regression, using a random intercept for promptID to account for prompts that used identical wording (because each LLM x verbal term x anxiety x severity phrasing was repeated 20x). This allowed us to examine between-prompt results rather than within-prompt variability.

- Numeric interpretations were analyzed with a two-way mixed ANOVA. Post-hoc testing using Tukey's honest significant difference test was performed when main effects were significant.

  Each LLMs' interpretation for each term was compared to human interpretations and the EC guidelines (both from Andreadis et al. 2021[1]) using one-sample t-tests.

eReference. Code Repository

https://github.com/nicholas-j-jackson/LLM-Medical-Prob

© 2025 Jackson NJ et al. *JAMA Network Open*.
