# Supplementary material for: Large Language Models and Communication of Medical Probabilities
Source: JAMA Netw Open. 2025 Dec 17;8(12):e2550449. doi: 10.1001/jamanetworkopen.2025.50449 (PMC12712724; doi:10.1001/jamanetworkopen.2025.50449)
Supplement: Supplement 2. — Data Sharing Statement [file jamanetwopen-e2550449-s002.pdf]

Data Sharing Statement

Jackson. Large Language Models and Inconsistent Communication of Medical Probabilities. *JAMA Netw Open*. Published December 17, 2025. doi:10.1001/jamanetworkopen.2025.50449

Data
Data available: Yes
Data types: Data (not involving human participants)
How to access data: The code necessary to reproduce this work and the results used in the paper are available at: https://github.com/nicholas-j-jackson/LLM-Medical-Prob
When available: With publication

Supporting Documents
Document types: Statistical/analytic code
How to access documents: https://github.com/nicholas-j-jackson/LLM-Medical-Prob
When available: With publication

Additional Information
Who can access the data: Anyone can access the code and the data
Types of analyses: For any purpose
Mechanisms of data availability: without investigator support or request
